# Anzansi family program: a study protocol for a combination intervention addressing developmental and health outcomes for adolescent girls at risk of unaccompanied migration

**DOI:** 10.1186/s40814-020-00737-4

**Published:** 2020-12-07

**Authors:** Ozge Sensoy Bahar, Fred M. Ssewamala, Abdallah Ibrahim, Alice Boateng, Proscovia Nabunya, Torsten B. Neilands, Emmanuel Asampong, Mary M. McKay

**Affiliations:** 1 Brown School, Washington University in St. Louis, Campus Box 1196, One Brookings Drive, St. Louis, MO 63130 USA; 2 School of Public Health, University of Ghana, Accra, Ghana; 3 Department of Social Work, University of Ghana, Accra, Ghana; 4 School of Medicine, University of California San Francisco, San Francisco, LA USA

**Keywords:** Child labor, Child independent migration, Adolescent girls, Intervention, Ghana

## Abstract

**Background:**

The International Labor Organization (ILO) estimates that 11% of children (ages 5 to 17) worldwide are child laborers. ILO recently drew attention to migrant child laborers as an underreported, but more vulnerable group to adverse outcomes relative to children working locally. Sub-Saharan Africa (SSA) continues to be the continent with the highest rates of child labor, with Ghana registering one of the highest incidence rates at 22%, including unaccompanied child migrants engaged in labor. Adolescent girls make up the majority of unaccompanied rural-to-urban migrants in search of better economic opportunities. Studies document the myriad of serious threats to health and emotional well-being experienced by adolescent girls who migrate to engage in child labor. These threats underline the urgent need for theoretically informed preventive interventions, specifically tailored to address the root causes of female child migrant labor and the needs of girls from economically insecure families and communities.

**Methods:**

A two-arm cluster randomized control trial will be conducted to assess the feasibility, acceptability, and preliminary impact of ANZANSI (family economic empowerment + multiple family groups) among 100 adolescent girls and their caregivers in the Northern Region of Ghana. Ten schools will be randomly selected from a list of eligible schools, and randomized to one of two study arms: (1) control arm (*n* = 5 schools, *n* = 50 adolescent-caregiver dyads); (2) treatment arm (*n* = 5 schools, *n* = 50 adolescent-caregiver dyads) receiving ANZANSI over a 9-month period. Adolescents (ages 11 to 14) in the same school will be assigned to the same study condition to avoid contamination.

**Discussion:**

The primary aim of the study is to address the urgent need for theoretically and empirically informed interventions that prevent adolescent girls’ unaccompanied rural-to-urban migration for child labor. Existing programs are not preventive and primarily target children who already migrated to the city and are living and working on the streets. This study is one of the first studies to pilot test a combination intervention, integrating family economic empowerment targeting household poverty with multiple family groups addressing family cohesion and perceptions on gender norms, child education/labor, all of which are factors, when combined, force girls to drop out of school and migrate.

**Trial registration:**

ClinicalTrials.gov; NCT04231669; Registered January 18, 2020;

## Introduction

An estimated 11% of children (ages 5-17) worldwide are child laborers and 41% of those are female [1, 2]. Sub-Saharan Africa (SSA) has the highest rates of child labor (22%) in the world [1]. Within SSA, Ghana has one of the highest rates of child labor where 21.8% of children engage in child labor [3, 4]. In addition to poor academic outcomes, child labor has serious health and mental health consequences for children [5–7]. Poverty across SSA is a factor that leads to unaccompanied rural-urban child migration and drives the high incidence of child labor [8]. An unaccompanied child migrant is “an individual below the age of 18, who has permanently or temporarily changed their place of residence without an adult guardian also migrating with them” [8]. Unaccompanied child migration and child labor are intricately related. Many unaccompanied migrant children need to work in order to financially survive [8].

### Female child labor and unaccompanied migration

In Ghana, girls are more likely to engage in hazardous child labor compared to boys [9]. Family commitment to girls’ education is often undermined in the face of limited financial resources [10]. Due to traditional gender norms in SSA, including Ghana, school attendance and graduation are rarely emphasized for girls. Emphasis is mainly placed on home directed outcomes (e.g., household chores, marriage, and childbearing) [11]. Consequently, school dropout, work, marriage, and childbearing are acceptable options for girls in the region [12]. Without education opportunities, adolescent girls living in poverty increasingly make up the majority of the North-South migrants in Ghana, mainly working as head-load carriers (Kayayei) [11, 12], a hazardous but attractive job for adolescent girls who have discontinued their education. Head load carrying (Kaya work) requires no capital investment [13], yet allows girls to immediately earn small amounts of money. Girls identify poverty, family, and education-related expenses, as key drivers for their unaccompanied migration to big cities [2, 10, 14]. More importantly, many girls engage in a cyclical pattern of migratory child labor, which puts them recurrently at risk [15].

### The impact of child unaccompanied migration, child labor on adolescent outcomes

Child labor has both immediate and long-term negative consequences for adolescents, including psychosocial well-being, mental health functioning, and educational trajectories [6, 16, 17]. Education is an essential means to acquire human capital to lift an individual/family from poverty [18]. Yet, children’s engagement in labor constitutes a significant threat to their education, hindering their chance to break the cycle of poverty [4, 18, 19]. This is even more critical for female children and adolescent girls as female education delays marriage, increases their chances to enter paid labor market, and improves health outcomes for females and their children when they become mothers (lower infant mortality, better nutrition) [20]. While child labor can be a critical household survival strategy, the abovementioned negative consequences make it a dubious long-term investment [12, 21]. However, few interventions have focused on reducing unaccompanied child migration, and to the best of our knowledge, none has focused on adolescent girls who are more susceptible to this high-risk situation [4].

### Families’ role in adolescent well-being and gender socialization: a need for family-focused interventions

Families are highly influential during adolescent development and play a critical role in potential interventions intended to meet the needs of adolescents [22, 23]. They foster children’s development through day-to-day counseling, directing children’s behavior in reference to standards or role expectations (including gender norms) communicated through interactions with the child [24]. The quality of the relationship between a child and his/her family can predict child adjustment [25, 26]. Youth with more frequent and open parent-child communication have better psychological adjustment and less susceptibility to peer pressure [22, 23]. Studies identified child maltreatment as a risk factor for unaccompanied child migration [27, 28]. Moreover, parental beliefs and attitudes on gender norms and education are closely related to parental decisions on child labor [29]. Hence, it is critical to closely work with families on strengthening family supportive processes and changing attitudes.

### Need for combination interventions

Kayayei are identified as a high-risk population, yet there is limited effort to prevent its occurrence. Recent child welfare efforts in Ghana have focused on addressing child trafficking, forced labor, and strengthening of policies and monitoring systems for children specifically working in the cocoa sector [4]. Some programs focus on providing school feeding programs or defraying the cost of education in public primary schools [4]. Other programs primarily target children who have already migrated to the city [4], ignoring the key drivers that lead to migrant child labor. While critically important, these programs have significant limitations. They tend to target one aspect of the problem and do not address the immediate needs of the families that lead to unaccompanied migration. The interconnected nature of risk factors leading to the unaccompanied migration of children and adolescents for work in big cities requires a comprehensive approach to prevention efforts [30], including combination interventions, to achieve greater impact—hence, the need for the ANZANSI family program.

This study titled ANZANSI family program (hereafter, ANZANSI, meaning resilience in the main local language in the Northern region of Ghana) focuses on adolescent girls before they drop out of school, but as they begin exhibiting early signs for dropping out (e.g., declining grades, skipping school and/or irregular attendance). Specifically, ANZANSI combines an evidence-informed family-level economic empowerment intervention aimed at creating and strengthening financial stability through the use of matched children savings accounts (CSA) and microfinance in households living in poverty, with a multiple family group intervention focused on addressing family functioning and parental beliefs around girls’ education, gender norms, and child labor. Informed by asset theory [31, 32] and parental ethnotheories framework [32, 33], the study uses a cluster randomized control trial to address the following specific aims:

#### Aim 1

Pilot test the (i) feasibility and acceptability of ANZANSI (family economic empowerment + multiple family group tailored to meet the specific needs of the targeted adolescent girls); and (ii) preliminary impact of ANZANSI by comparing the control arm to the treatment arm on specific child development outcomes, including school attendance; intention to independently migrate; family financial stability; psychosocial and mental health functioning; and family cohesion.

#### Aim 2

Explore multi-level factors (individual, family, and programmatic) impacting participation in and experiences with the ANZANSI (feasibility, acceptability, facilitators, barriers, and recommendations).

### ANZANSI family program

This intervention combines two evidence-based interventions tested in the sub-Saharan Africa: Family economic empowerment intervention tested in Uganda and Kenya, and multiple family group intervention tested in Uganda, Kenya, and Ghana.

#### Family economic empowerment

This component includes (1) workshops on asset building, future planning, and protection from risks; (2) child development account (CDA); and (3) family income-generating/microenterprise promotion component.

##### Workshops on asset building, future planning, and protection from risk

Twelve 1-2 h workshop sessions on (a) asset building and the means through which asset building occurs; (b) income generating options; (c) short- and long-term education, career and life goals; and (d) saving and ties to financial institutions.

##### CDA

Each participant receives a CDA, a matched savings account held in the adolescent girl’s name and her caregiver as a co-signer, in a formal financial institution. Participants’ family members, relatives, or friends are allowed and encouraged to contribute toward the CDA. The account is then matched with money from the project. The match cap (the maximum amount of family contribution to be matched by the intervention) is an equivalent of US$10 per month per family or US$90 for the 9-months intervention period. SUUBI studies in Kenya and Uganda indicate that families can save these amounts and that the amount is adequate and reinforcing [34–36].

During the intervention, every equivalent of $1 saved (in Ghana currency) by the participant and her family, will be matched by $2 from the project (match rate of 1:2). Thus, adolescent girls who save the maximum amount will have $270 at the end of the intervention ($90 in their own savings plus $180 from the match). This money can be used to keep the adolescent girl in school (e.g., pay for school uniform, books, supplies) and/or start a small business for her and her family outside of school hours. During the intervention, each girl, with her primary caregiver as a co-signer, has access to the money in her account (excluding the matching funds). Participants can withdraw their own money (but not the matching funds) only in case of emergency. When ready to use their match for business expenses, participants meet with the study team to review their business plan and supply costs. A check for the matching funds is then written directly to the school or “the vendor,” supplier of the business capital items.

##### Family income-generating activities

Participants are trained on income-generating activities and expected to use part of their matched savings to start an income-generating activity intended to benefit the whole family.

#### Multiple family groups

Rooted in family systems theory, structural family theory and social learning theory with elements of psychoeducation and social group work [24, 25], multiple family group intervention is a family-centered, group-delivered, and evidence-informed intervention designed for children and adolescents whose families struggle with poverty and associated stressors, which is highly relevant to the proposed study context [25]. This intervention has been applied to a range of target populations struggling with a diverse range of issues in the USA as well as sub-Saharan Africa (South Africa, Uganda, Ghana, and Kenya). The intervention is based on building family support through opportunities for parents and children to communicate in a safe setting with other families who have shared experiences and allow each family to learn from one another [25, 37]. The multiple family group intervention recognizes that poverty-related factors may undermine parenting [37], and in this case, may contribute to decisions for adolescent girls to migrate for work.

More specifically, multiple family group intervention involves six to eight families in the USA and 12 to 20 families in South Africa [25, 37]. At least two generations of a family are present in each session. Content and practice activities foster learning and interaction both within families and among families in the sessions [25]. The intervention targets primary school-age children, ages 7 to 13 years [25, 38]. Children and their families (including adult caregivers and siblings over six years of age) are invited to attend 16 sessions. The sessions combine group discussions and activities such as roleplays. While children and caregivers may split to complete some activities within the sessions separately, they reconvene for sharing and discussing as a larger group. The skills and family processes referred to as the 4Rs (Rules, Responsibility, Relationships, and Respectful Communication) and 2Ss (Stress and Social Support) organize the curriculum [25]. ANZANSI will utilize the manualized multiple family group intervention already adapted to the Ghana context as part of another project (SMART Africa Center, U19MH110001) and will tailor it to the needs of this population. More specifically, the intervention will include discussions on child roles, gender norms, and child labor.

We expect that incentivizing families to keep their adolescent girls in school via economic empowerment and multiple family group interventions may be more effective in impacting gender norms and perceptions on child labor, specifically the value of education for girls. As school expenses and gender norms are significant barriers to school attendance and completion, and a factor in prioritizing education for boys over girls, creating family-level assets targeted to cover school expenses for girls while encouraging families to discuss children’s roles, gender norms, and risks associated with child labor are critical to ensuring equal opportunities for girls.

### Theoretical frameworks

#### Asset theory

Asset theory posits that assets (e.g., family financial resources, access to protected income-generating opportunities, including family business) improve family stability and have important developmental and psychological benefits for children and adolescents [31, 39, 40]. Asset building refers to efforts that enable people with limited resources to acquire and accumulate long-term productive assets [31, 39]. It is increasingly viewed as a critical poverty reduction strategy, positively impacting attitudes and behaviors, and improving psychosocial functioning and stability [34, 35, 40]. Increased assets lead to expectations for more resources in the future, realistic optimism, feelings of safety and security, and future planning [34, 35, 40]. Asset theory would predict that an adolescent girl with no means to support her education and no belief that her future holds promise is more likely to skip school, drop out, and migrate independently to look for alternative means of earning. Unfortunately, with no employable skills, she is likely to engage in hazardous work (e.g., Kayayei in Ghana), exposing her to multiple risks. However, if the adolescent girl is provided with the economic support to stay in school, and her family achieves a sense of financial stability, their outlook for the future may change. She may stay in school, get good grades, and build human capital for future employment. ANZANSI is expected to increase household economic stability, provide a financially viable route for adulthood, and influence the decision for girls to stay in school.

#### Parental ethnotheories framework

Parents’ understanding of gender norms, child education, and child labor are developed within a particular culture, community, place, and time, and are called “parental ethnotheories” [32, 33]. How parents conceptualize gender norms, child education, and child labor is a reflection of parental socialization, which influences parental actions and decisions about children and their development in systematic ways [32]. Parental socialization is important in providing parents with knowledge, not only about how children become functional members of society [32] but also expectations regarding children’s contribution to everyday family life. Although this framework may have some universal dimensions, it is constructed within cultural belief systems and socialization, and it is often implicit or taken for granted as ideas that have strong motivational properties for parents [41]. Thus, in addition to improving family cohesion, the multiple family group component of ANZANSI is intended to help families to shift existing misconceptions about gender, education, and labor.

## Methods

**Fig. 1:**
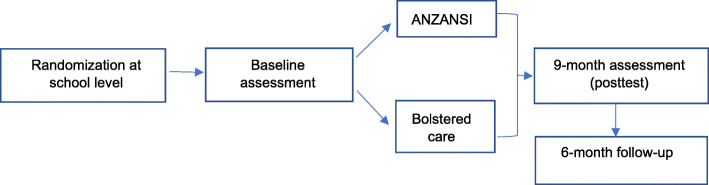
Anzansi design

We propose a two-arm cluster randomized control trial evaluating the feasibility, acceptability, and preliminary impact of ANZANSI (family economic empowerment + multiple family groups) among 100 adolescent girls and their caregivers. Specifically, this RCT aims to (1) refine and pilot test ANZANSI’s feasibility, acceptability, and study procedures (recruitment, retention, data collection); (2) identify barriers and facilitators to ANZANSI’s implementation; (3) estimate intervention parameters (e.g., study population variance, attrition, and response rates); and (4) examine the preliminary impact of ANZANSI (e.g., effect size) on identified outcomes for adolescent girls and their families, post-intervention initiation at 9 months (posttest), and 15 months (6-month follow-up) (see Fig. 1).

Ten schools will be randomly selected from a list of eligible schools, and randomized to one of two study arms: (1) control arm (*n* = 5 schools, *n* = 50 adolescent-caregiver dyads): (2) treatment arm (*n* = 5 schools, *n* = 50 adolescent-caregiver dyads) receiving ANZANSI (family economic empowerment + multiple family groups) over a 9-month period. Adolescent girls in the same school will be assigned to the same study condition to avoid contamination.

We hypothesize that following the intervention, compared to the control arm, participants receiving the ANZANSI intervention (treatment arm) will have (a) improved school attendance and attitudes toward schooling; (b) lower mean levels of intentions to migrate; and (c) improved psychosocial well-being, social support, and family functioning.

### Study setting

The study will be conducted in the northern region of Ghana, which is one of the three poorest regions in the country, with the highest incidence (81%) of multi-dimensional poverty index, highest rates (35%) of child labor, and lowest rates of school attendance in the country [42–45]. In the region, only 22% of the population age 15 years and older are classified as literate [46, 47]. Specific vulnerabilities relating to young women and girls in the region include traditional gender norms, early and forced marriages, and higher rates of school dropout compared to their male counterparts [47], and living in one of the main sending regions for unaccompanied adolescent girls to major cities for work.

Schools within the Tamale District with the highest rates of female student dropouts at the middle school level (based on retrospective 3-year data) will be compiled. Once the list is compiled, the ten schools with the highest female student dropout rates will be selected. Subsequently, schools will be randomly assigned to either control or treatment arm. Following school randomization, the study team will work closely with the schools for recruitment.

### Participants

One hundred adolescent girls and their caregivers will participate in the study (*n* = 50 dyads in each arm; 10 per school). Adolescent girls’ inclusion criteria are (1) enrolled in school and living within a family (defined broadly—not necessarily biological parents); (2) ages 11 to 14; (3) capable of giving assent; and (4) skipping school in the past academic term (with at least 10% of unexcused absences). The caregiver inclusion criteria are (1) self-identified as primary caregiver of the adolescent girl and (2) capable of providing informed consent. Participants (girls and caregivers) that do not meet the criteria or exhibit a lack of understanding of the study procedures and hence not able to provide informed consent will be excluded.

### Recruitment, retention, and attrition

School administrators will share a flyer that introduces the project to the students and caregivers; and invite all the caregivers with an eligible child/ren to contact the school for details. The study team will organize meetings with families in each of the selected schools to present the study and answer questions. The research team will then meet with potential participants to engage them in the screening and informed consent process. Adolescent girls will be contacted for assent only after receiving consent from their caregivers. Only families who provide both consent and assent will be recruited to participate in the study.

### Study arms

#### Control arm (bolstered care)

Adolescent girls in the bolstered care will receive services/education as usual in their respective schools. The usual care will be bolstered by providing school notebooks and lunch in the control arm (bolstered care will also be provided to the treatment arm). Primary school education is universal and free in Ghana. Yet notebooks and lunch are costly expenses for families that create a barrier to school attendance. Hence, these will be provided to participants in all study schools.

#### Treatment arm (ANZANSI)

In addition to bolstered care, participants in this arm will receive the ANZANSI intervention described earlier.

#### Intervention adaptation, delivery, monitoring, and fidelity

Community stakeholders will be gathered and engaged in tailoring the content of the existing multiple family group manual focused on child behavioral health used in Ghana. BasicNeeds (implementation partner for the proposed study) staff already trained in this intervention delivery used for SMART Africa will attend a refresher training before training facilitators to deliver this part of ANZANSI. Ten female facilitators will be selected from the community. Core components of the multiple family group intervention will be maintained. Intervention sessions will occur via 1-h group meetings facilitated by two facilitators. Adapted protocols will guide the program delivery [48, 49]. Independent observations will be made in a random sample of 60% of intervention sessions using fidelity checklists. These observations will be used to assess the planned versus actual implementation; evaluate implementation integrity; and examine how strategies were altered to maximize effectiveness and acceptability.

#### Implementation process evaluation

Changes from the planned curriculum will be examined using process notes. Caregivers will report monthly on factors that affect their participation using an implementation checklist that assesses satisfaction and obstacles to program delivery: (1) factors interfering with adolescent girls’ or family participation (e.g., insufficient time, health, stigma, competing priorities); (2) concrete obstacles (e.g., weather, transportation, sickness); and (3) site and staffing obstacles (e.g., time and space constraints). Caregivers and adolescent girls will also complete a short checklist on barriers/motivators developed for “Knowledge about the African American Research Experience” (R01MH58566; PI: McKay).

#### Quantitative data collection

Data will be collected at baseline and post-test (9 and 15 months post-intervention initiation) from both adolescent girls and their caregivers (see Table 1 for measures).

**Table 1:** Measures

***Variable***	***Measures***
**Demographics (A, C)**	Demographic survey
**School (A, C, S)**	1) School Attitude Assessment Survey-Revised [50] 2) School attendance reports
**Work attitudes (C)**	Attitudes toward youth employment [51]
**Savings (A, C, B)**	1) Bank statements 2) Importance of savings 3) Confidence of savings 4) Financial literacy
**Psychosocial well-being (A)**	1) Tennessee Self-concept Scale Short Form [52] 2) Rosenberg Self-esteem Scale [53] **3) Emotional Self-Efficacy Scale** [54] 4) Social Self-Efficacy Scale [55] 5) Multidimensional Student Life Satisfaction Scale [56] 6**) Adolescent Stress Questionnaire** [57, 58]
**Family/social support (A, C)**	1) Social Support Behavior Scale [59] 2**) Multidimensional Scale of Perceived Social Support** [60]
**Family relations (A, C)**	1) Family Environment Scale (FES) [61–63] 2) Family Assessment Measures (FAM) [61–63]
**Migration (A, C)**	How likely they are to see themselves migrate? How likely they are to send their daughters to work? Follow-up (6 months) are girls working?
**Gender attitudes (A, C)**	1) Attitudes toward women [64] 2) Gender Norm Attitudes Scale [65]
**Future orientation (A)**	Thinking about the future [42]
**Feasibility**	Recruitment rates; staff level of effort; number of screenings conducted; proportion eligible and agreed to enroll**; number of rescheduled, canceled, missed sessions/ assessments to inform staffing needs and retention protocols for subsequent trial
**Acceptability (A, C)**	Client Satisfaction Questionnaire (CSQ-8; to be adapted) [66] Semi-structured interviews

*A* adolescent, *C* caregiver, *B* bank

**Enrollment of 70% or higher will be considered feasible [67]

Measures that have been used in SSA are underlined

Measures that have been used in Ghana are in bold

The complete assessment battery is administered at each timepoint to inform a future R01 level study, should findings warrant. The battery takes into account: (1) participant’s literacy (read aloud procedures); (2) need for trust and rapport; and (3) use of local phrases/terms. Adolescents will be fluent in English, yet caregivers may be only speaking Dagbani (local language). Thus, all research assistants (RA) will be fluent in English and Dagbani, and assessments will be available in both languages (using forward/backward translation by a certified translator). The in-country Multiple Principal Investigator (MPI) Ibrahim is fluent in Dagbani and will help cross check the translated documents. The assessments will be conducted by trained RAs in a private location and take approximately 60 min with a 10-min break. Content and construct validity will be conducted for measures to ensure cultural validity where necessary.

### Qualitative data collection

Upon intervention completion, 30 randomly selected dyads from the treatment arm will be invited to participate in interviews about their experiences with the ANZANSI intervention. We expect that this sample size will be sufficient in reaching theoretical saturation [68, 69]. Questions will be translated and back translated by 2 team members fluent in both English and Dagbani (local language). The interviews will be conducted with caregivers and adolescent girls separately in a private place of their choosing by RAs (trained in qualitative interviewing) using pre-established semi-structured interview protocols. The interviews will focus on (1) experiences with the intervention; and (2) key multi-level (individual, family, contextual, and program) influences affecting program participation. Semi-structured interviews elicit richer responses [70] and will help better understand the following: (1) participants’ processes and experiences with ANZANSI; (2) potential processes behind key outcomes and mediating variables; and (3) multi-level factors that may have affected their experiences. Interviews will last 1 h and will be audio taped. The interviews will assess intervention feasibility and acceptability, not children’s psychosocial functioning. Hence, we do not expect the interviews to influence follow-up assessment.

### Data analysis

#### Feasibility

We will monitor recruitment rates and staff’s level of effort, as well as the number of screenings conducted, proportion eligible, and agreed to enroll. Enrollment of 70% or higher will be considered feasible [71]. We will also record the number of rescheduled, canceled, and missed sessions and assessments to inform estimation of staffing needs and retention protocols for future trials.

#### Acceptability

We will adapt the Client Satisfaction Questionnaire (CSQ) to assess acceptability [66]. Items include “How helpful was ANZANSI in addressing school dropout and intention to migrate to urban centers to work?” and “How likely are you to recommend ANZANSI to families whose children are at risk of migrating to urban centers to engage in child labor?” Given the modest sample size, quantitative analyses of intervention data will be largely descriptive and concentrate on tabulating and summarizing satisfaction outcomes.

#### Preliminary impact

We expect that following intervention, relative to the control arm, participants in ANZANSI will have (a) improved school attendance and attitudes toward schooling; (b) lower mean levels of intentions to migrate; (c) improved psychosocial well-being, social support, and family functioning.

Children will be the units of analysis for the preliminary analyses. We will plot means by group over time to describe overall patterns of change. We will use linear mixed models (LMMs) to evaluate the proposed hypotheses. We will fit LMMs to ensure that all requisite information is available to perform the types of analyses typically undertaken in a formal RCT of intervention efficacy and to obtain valuable effect size information. LMMs will include random intercepts for school membership, and random intercepts and slopes for subjects (three-level models).

Additional exploratory analyses will study caregivers and children jointly as the unit of analysis via dyadic analysis methods, such as actor-partner and means-and-deviation models, to quantify caregiver vs. child effects and between- vs. within-dyad effects on psychological well-being [71, 72].

#### Power calculations

Although the study purpose is to determine preliminary feasibility and acceptability rather than conduct formal hypothesis tests, we conducted several power analyses using NCSS PASS to supply additional information. Our power analyses assume *α* = 0.05, power = 0.80, 83 participants retained at the final time point following 17% estimated attrition, and a conservative unconditional intra-class correlation coefficient (ICC) of 9.3% based on our previous SUUBI study of school-going children and school attendance in Uganda. For the target enrollment proportion of 70% to assess feasibility, the width to the limit of the confidence interval is 24.4% (standardized distance: 0.28). For continuous standard normal variables to assess acceptability (e.g., CSQ-8), the distance from the mean to the confidence limit is 0.26. These distances to confidence limits are between small (0.20) and medium (0.50) effect sizes. For preliminary efficacy exploratory analyses with two post-baseline time points, minimum detectable standardized mean differences ranged from 0.56 to 0.69 for within-subjects correlations ranging from 0.20 to 0.80. In sum, our study is powered to detect small to medium distances to confidence limits for descriptive statistics and medium to large longitudinal analysis effects depending on the amount of within-subject correlation of responses, though, as noted previously, hypothesis testing is not the study focus.

#### Handling of missing data

We will rely on direct maximum likelihood estimation (MLE) or a multiple imputation strategy [73] to accommodate missing data, when necessary. Inclusion of cases with partial data is an important step since an intention-to-treat approach is planned to be the main analytic strategy for the primary aim focused on generating preliminary estimates of ANZANSI’s efficacy. If statistical and/or clinical significance of treatment effects appear to differ depending on how missing data are addressed in analyses, such findings will be reported.

#### Qualitative data analysis

Interviews will be transcribed verbatim and translated where necessary. All transcripts will be uploaded to the NVivo12 analytic software. Transcripts will be reviewed by the research team to have a broad understanding of the content and identify topics for discussion/observation. Analytic induction techniques will be used for coding [74–77]. Initially, 5 randomly selected interview transcripts will be read multiple times and independently coded by the team using sensitizing concepts and identifying emergent themes (open coding) [74–77]. Themes will be broken down into smaller, more specific units until no further subcategory is necessary. Potential themes include barriers and facilitators at the individual (e.g., time constraints, interest); family (e.g., competing demands, support); program (e.g., content relevance; interaction with other families); and contextual levels (e.g., cultural norms). Analytic memos will be used to further develop themes/subthemes, and integrate ideas emerging from the data. Codes and the inclusion/exclusion criteria for code assignment [78] will be discussed as a team to finalize the codebook. Transcripts will then be coded by two investigators independently and inter-coder reliability will be established. A level of agreement between 66-97% indicates good reliability [78]. Disagreements will be resolved through team discussions. The secondary analysis will compare themes across participants, and between caregivers and adolescent girls for similarities, differences, and relationships. Member checking, peer debriefing, and audit trail will be used for rigor [70].

### Data safety and monitoring

To protect the integrity of the participants’ data, the following standard procedures will be followed. First, the data collected from the study participants will be used only for the purpose of research. All data will be kept confidential. Second, all families (adolescent girls and caregivers) participating in the study will be assigned a random ID number by the in-country MPIs (Drs. Ibrahim and Boateng) and the research team. This ID number will be used on all information collected from participants, including questionnaires and audiotapes. Since the study has multiple data collection points, we will maintain lists of participants with links between identifying information and ID numbers. Only the PI (Sensoy Bahar), in-country MPIs, Project Director, and Research Assistant will have access to these lists, which are kept in locked files.

All personnel must complete the CITI Human Subjects and Good Clinical Practice Training certifications, and sign confidentiality statements that specify that if the participants’ confidentiality is breached unintentionally that personnel will follow the procedures for reporting this breach to the principal investigator (PI). Research personnel also participate in training with the PI, in-country MPIs, and/or Project Director regarding data safety, confidentiality of participants, limits of confidentiality, and proper administration of the study protocol.

All hard copies of data are stored in locked cabinets to which only the PI, in-country MPIs, Project Directors, and Research Assistants have access. After completion of an interview with a study participant, data with ID numbers is placed in a separate locked file cabinet while waiting for entry. Once data is entered into computer files and password protected, only the PI, in-country MPIs, Project Directors, and data entry assistants have access to these files. Consent forms will be stored in a different locked cabinet separately from any notes and other paper-based data to further insure confidentiality. Audio taped interviews will be transcribed by authorized individuals only, and participants will be identified only by their assigned ID numbers which are recorded on all data including tapes. Typed transcriptions will be stored electronically on password-protected computers only. All requests, current and future, to use the data will be reviewed by the PI and in-country MPIs. Any data files that are provided to other individuals will be stripped of identifiers and contain only ID numbers so that data across multiple assessment waves can be matched.

Within the informed consent process, participants and caregivers are notified of the above procedures. Participants are also informed of the limits of confidentiality. Specifically, the exception to confidentiality should there be risk of immediate harm to the child or others. For example, if a child tells us that an adult is abusing her, or that she is abusing a child, or that she is going to hurt herself or someone else, in such circumstances, the answers will be shared with the local authorities. All these safeguards and potential risks will be explicitly stated in the assent and consent forms, and explicitly explained to the participants and their caregivers.

To ensure that interviewers have accurate knowledge of what does and does not constitute reportable child abuse and/or neglect, interviewers will receive training on the Ghanaian child protection laws. Interviewers who suspect child abuse and/or neglect will be instructed to contact the in-country MPIs and/or PI, rather than contact the local authorities themselves. Prior to making a decision of whether to make a report of child abuse and/or neglect, the case will be discussed among the group consisting of the interviewer, the in-country MPIs, and PI. If we determine that a report must be made, we will inform the caregiver of our intention to report and provide reasons why a report must be made, unless we think that doing so would pose an immediate risk to the adolescent girls. The study will maintain records of adverse events, any referrals for counseling, as well as copies of the consent and assent forms. All records will be maintained in a locked filing cabinet at the University of Ghana School of Public Health office accessible only by the research team. The PI and MPIs will be responsible for data security and record-keeping. The data sets used for analysis will not contain any identifying information—specifically, names and addresses of the participants.

### Plan for disposition of identifiers at the end of the study

Identifiers for the participants will be disposed of not more than 3 years after study completion. To protect the participants’ confidentiality, identifiers only will be accessible by the PI and the in-country MPIs, and will be kept separately from other participants’ responses.

### Protection from potential safety/clinical risks

The study will institute important safeguards to protect the welfare of study participants. The PI will train team members to identify risk factors associated with adverse events. For purposes of this study, adverse events include potential for suicide, homicide, worsening of participant physical health, new or escalating physical/emotional abuse occurring within families, or worsening of participant physical health that may be related to the proposed intervention. The research team will be instructed that if any adverse event or risk related to a child or caregiver involved in the study is identified, participant involvement will be halted immediately and the appropriate personnel contacted. Study staff will be informed of the protocol of rescue procedures in the occurrence of adverse events, which begins with the notification of the in-country MPIs via phone and email. The in-country MPIs are very knowledgeable about existing resources that can provide help and support to participants in the study that experience clinical emergencies or need additional interventions that cannot be provided within the research study context. In addition to the in-country MPIs, the research staff will also immediately contact the PI via phone and email once the MPIs in Ghana have been notified.

### Monitoring and responding to adverse events

All study personnel based in Ghana will be trained in identifying indicators of conditions that may jeopardize the welfare of participants and the limits of confidentiality. This training includes reviewing possible scenarios and knowledge of key questions used to assess risk. Interview staff are trained to err on the side of caution and told to contact the in-country MPIs, who will always be available, by telephone, in the event of the need to break confidentiality due to mandatory reporting or ethical concerns. Under the guidance of in-country MPIs, research staff are trained either to contact the police to ensure safety of participants, or if appropriate, to have emergency personnel take the adolescent girl or caregiver to the nearest hospital.

In the case of an adverse event, staff will inform the in-country MPIs and then the PI within 24 h of the presence of a possible unanticipated adverse event. Any presence of a possible unanticipated adverse event will be immediately reported and brought to the attention of the Washington University Institutional Review Board (along with local IRB). The IRBs will determine whether it is appropriate to stop the study protocol temporarily or will provide suggestions and/or modifications to the study procedures. Possible modifications may include adding new risks to the consent form and re-consenting all study participants.

Preliminary outcome data will be examined quarterly by the PI and the in-country MPIs. If preliminary outcome data indicates harmful impact of the program to the study participants, the Washington University IRB committee, as well as the Ghana Health Service Ethics Review Committee will be notified and it is possible that the study will be discontinued immediately. However, we do not anticipate any negative effects of participating at this time as much of this program to be implemented will be based on local and cultural expertise.

## Discussion

The primary aim of the study is to address the urgent need for theoretically and empirically informed interventions that prevent adolescent girls’ unaccompanied rural-to-urban migration for child labor. More specifically, the study proposes to refine and pilot-test an innovative combination intervention to curtail poverty-impacted adolescent girls’ rural-to-urban migratory patterns and involvement in child labor in SSA, and consequently reduce their exposure to work-related risk factors undermining their health, well-being, and ultimately future.

Existing programs (mostly offered by NGOs) are not preventive and primarily target children/adolescents who already migrated to the city and are living and working on the streets, ignoring the key drivers that lead to migrant child labor and all its associated negative consequences. The study is innovative in several ways. It addresses the serious need for research and theory informed, gender specific, and contextually relevant preventive interventions targeting girl child laborers in LMICs. It is one of the first studies to pilot test a combination intervention, integrating family economic empowerment targeting household poverty with multiple family group intervention addressing family cohesion and perceptions on gender norms, and child education/labor, all of which are factors, when combined, force girls to drop out of school and migrate. We attempt to stabilize and positively impact school-going girls’ household financial situation, family relations, engagement in school, and to address cultural and gender norms on education. Moreover, the ANZANSI family program will be one of the few interventions to focus on adolescent girls at risk of unaccompanied migration for labor, recognizing the unique developmental and gender-specific needs and risks, and the implications of cultural norms (e.g., daughters to care for parents, child marriage) while also working together with their families. The delivery format for multiple family group intervention is culturally consistent with the SSA’s (and Ghana) collective approach of supporting and raising children. The cultural/familial norms are such that families raise children “together,” an approach akin to the multiple family group intervention, which strengthens its appeal to communities and likelihood of success.

Study findings will be disseminated through local, national, and global meetings and publications to facilitate sustainability and scalability. The research team will also hold stakeholder meetings to share the trial results with the communities affected by adolescent girls’ unaccompanied migration for labor.

Our study builds on collaborative partnerships with local investigators and stakeholders, which increases the understanding of the needs of the girls and their families; embeds the intervention in the community; and improves its chances of feasibility and acceptability, and the potential to conduct a R01-level RCT if results warrant. As several developing countries, including those in SSA, grapple with child labor, this study will lay the foundation for a larger intervention study investigating how the children’s unaccompanied migration for labor can be prevented.

## Data Availability

Not applicable.

## Abbreviations

CDA: Child Development Account
Co-I: Co-investigator
CSQ: Client Satisfaction Questionnaire
ICC: Intraclass correlation coefficient
ILO: International Labor Organization
IRB: Institutional Review Board
LMICs: Low- and middle-income countries
LMM: Linear mixed models
MPI: Multiple principal investigator
NGO: Non-governmental organization
PI: Principal investigator
RA: Research assistant
RCT: Randomized controlled trial
SSA: Sub-Saharan Africa
